# First Presentation With Impending Cardiac Tamponade Leading to a New Diagnosis of Mediastinal Lymphoma: A Shared Learning Case on the Atypical Presentation of Pericardial Effusion and Cardiac Tamponade

**DOI:** 10.7759/cureus.116816

**Published:** 2026-09-24

**Authors:** Samia Israr, Nihinsa S Ilandari Dewa, Michelle E Teo

**Affiliations:** 1 General Medicine, Hinchingbrooke Hospital, Huntingdon, GBR; 2 Internal Medicine, Hinchingbrooke Hospital, Huntingdon, GBR

**Keywords:** cardiac tamponade, diffuse large b-cell lymphoma, emergency pericardiocentesis, mediastinal lymphoma, oncological emergency, pericardial effusion, primary mediastinal b-cell lymphoma, r-epoch, superior mediastinal mass, young woman oncology

## Abstract

Cardiac tamponade is a life-threatening emergency classically associated with Beck’s triad of hypotension, muffled heart sounds, and elevated jugular venous pressure; however, incomplete or atypical presentations may delay diagnosis. We report the case of a previously healthy 28-year-old woman who presented with progressive dyspnoea, pleuritic chest pain, orthopnoea, peripheral oedema, fever, and fatigue. Examination revealed mild tachycardia, soft heart sounds, reduced basal air entry, and ankle oedema, while the patient remained normotensive. Initial investigations demonstrated cardiomegaly with pleural effusions on chest radiograph, low-voltage QRS complexes on electrocardiogram, and markedly raised inflammatory markers. Computed tomography pulmonary angiography identified a large anterior mediastinal mass encasing the great vessels, a significant pericardial effusion with features of cardiac tamponade, and bilateral pleural effusions. Emergency pericardiocentesis resulted in immediate clinical improvement. Histopathological analysis confirmed high-grade B-cell lymphoma, most consistent with primary mediastinal B-cell lymphoma. The patient was treated with immunochemotherapy, achieving complete metabolic remission on end-of-treatment PET-CT. This case highlights the importance of recognising atypical presentations of cardiac tamponade and considering mediastinal malignancy in young patients presenting with unexplained cardiorespiratory symptoms.

## Introduction

Primary mediastinal B-cell lymphoma (PMBCL) is an uncommon and aggressive subtype of large B-cell lymphoma that predominantly affects young adults and typically presents as a bulky anterior mediastinal mass [1,2]. Cardiovascular complications, particularly pericardial involvement and cardiac tamponade, are uncommon but potentially life-threatening manifestations of lymphoma [3]. PMBCL has distinct clinical and pathological characteristics and commonly presents with symptoms related to mediastinal compression, including dyspnoea, chest pain, cough, and superior vena cava obstruction [4,5].

Although pericardial involvement is recognised in lymphoma, cardiac tamponade as the initial presentation of PMBCL is rare. We present a case of a previously healthy 28-year-old woman who presented with progressive cardiorespiratory symptoms and was found to have a large anterior mediastinal mass, significant pericardial effusion, and radiological features of cardiac tamponade. This case highlights the importance of recognising atypical presentations of PMBCL and maintaining a high index of suspicion for significant pericardial disease despite the absence of classical clinical features of cardiac tamponade.

## Case presentation

A previously well 28-year-old woman presented with a one-week history of progressive dyspnoea, pleuritic chest pain, orthopnoea, paroxysmal nocturnal dyspnoea, peripheral oedema, fever, and fatigue. She had no significant past medical history.

On examination, she was mildly tachycardic but haemodynamically stable, with preserved blood pressure. Heart sounds were soft, and reduced air entry was noted at both lung bases. Bilateral ankle oedema was present, although jugular venous pressure was not clearly elevated.

Initial investigations demonstrated cardiomegaly with bilateral pleural effusions on chest radiography (Figure 1). Electrocardiography demonstrated low-voltage QRS complexes, which, in the context of cardiomegaly on chest radiography, raised suspicion of a significant pericardial effusion (Figure 2). Blood tests showed markedly elevated inflammatory markers, an elevated D-dimer level, mild lymphopenia, and raised alanine aminotransferase (ALT) levels (Table 1). The initial differential diagnoses included pulmonary embolism, pericarditis, and myocarditis.

**Figure 1:**
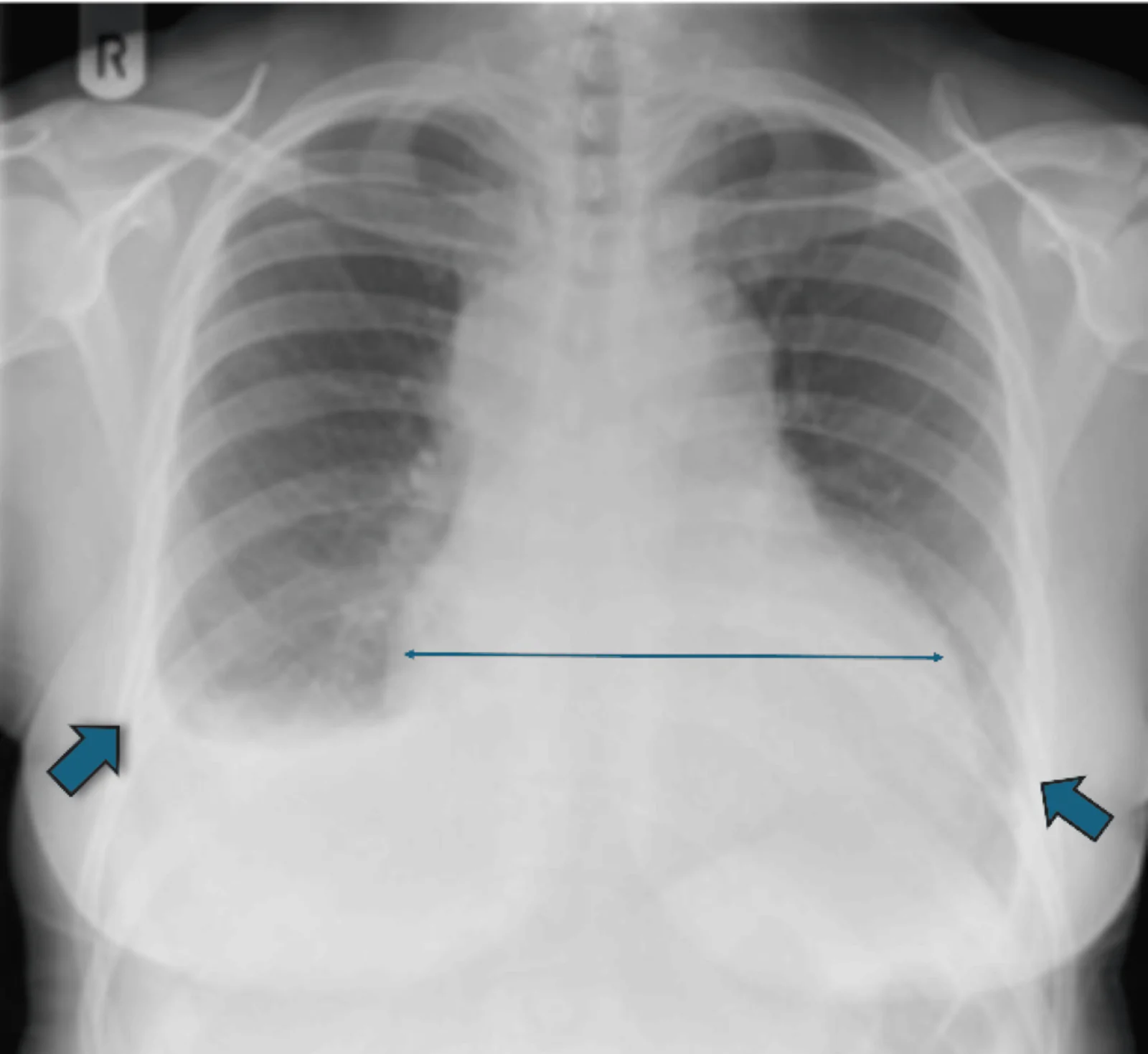
Admission chest radiograph demonstrating cardiomegaly and bilateral pleural effusions (arrows).

**Figure 2:**
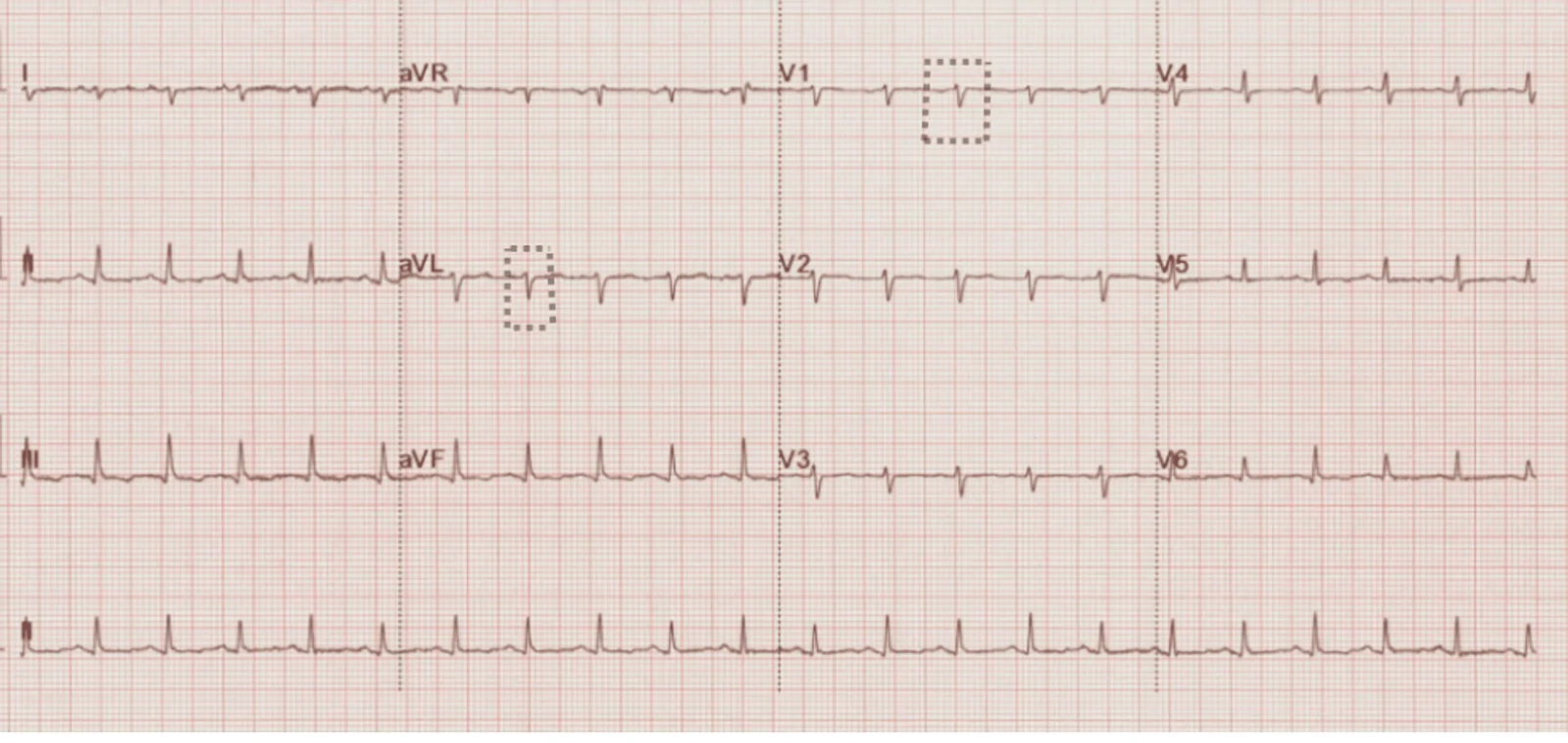
Admission ECG showing low-voltage QRS complexes as indicated in boxes. Low-voltage QRS on an ECG means the electrical signals from the heart are smaller than normal, specifically measuring less than 5 mm in all limb leads and less than 10 mm in all precordial leads.

**Table 1:** Relevant admission laboratory investigations.

Parameter	Patient value	Reference range	Unit
C-reactive protein (CRP)	66	<5	mg/L
D-dimer	3594	0-250	ng/mL
White cell count	9.8	4.0-11.0	× 10⁹/L
Lymphocyte count	0.8	1.4-4.8	× 10⁹/L
Haemoglobin	124	115-165	g/L
Platelet count	314	150-400	× 10⁹/L
Alanine aminotransferase (ALT)	134	<33	IU/L
Sodium	132	133-146	mmol/L

Urgent computed tomography pulmonary angiography revealed a large anterior mediastinal mass encasing the great vessels with impaired right pulmonary venous return. A large pericardial effusion with radiological features of cardiac tamponade was identified, together with bilateral pleural effusions (Figure 3).

**Figure 3:**
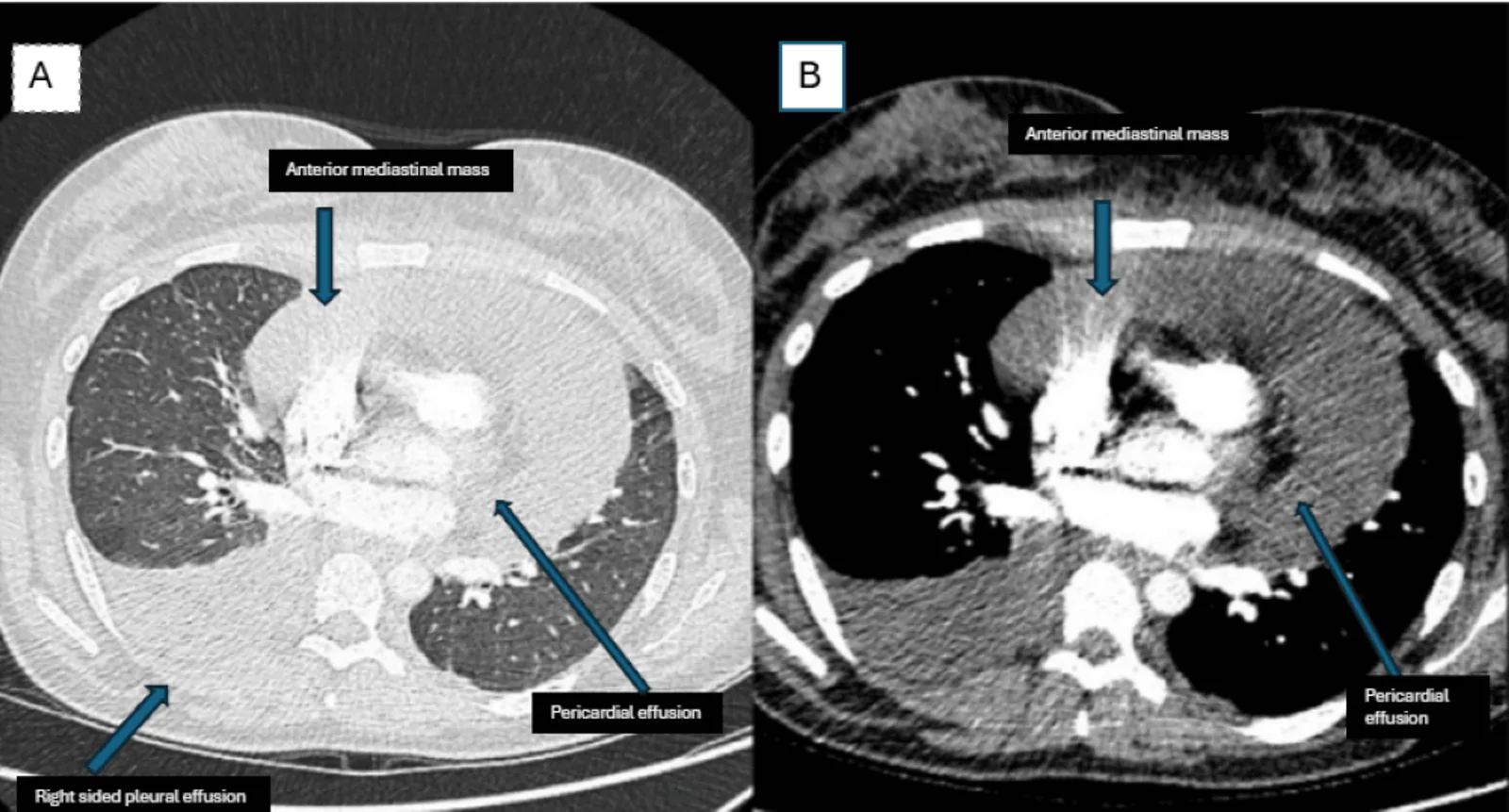
Computed tomography demonstrating a large circumferential pericardial effusion with large anterior mediastinal mass and pericardial effusion. A) Non-contrast computed tomography demonstrating the anterior mediastinal mass, right-sided pleural effusion, and circumferential pericardial effusion (arrows). (B) Contrast-enhanced computed tomography demonstrating the pericardial effusion with compression of the right-sided cardiac chambers and right-sided pleural effusion (arrows).

The patient was transferred urgently to a tertiary cardiothoracic centre, where emergency pericardiocentesis drained approximately 1.5 L of pericardial fluid, resulting in significant symptomatic improvement and interval reduction in cardiac silhouette on post-procedural chest radiography (Figure 4). Subsequent biopsy of the mediastinal mass confirmed high-grade B-cell lymphoma, most consistent with PMBCL.

**Figure 4:**
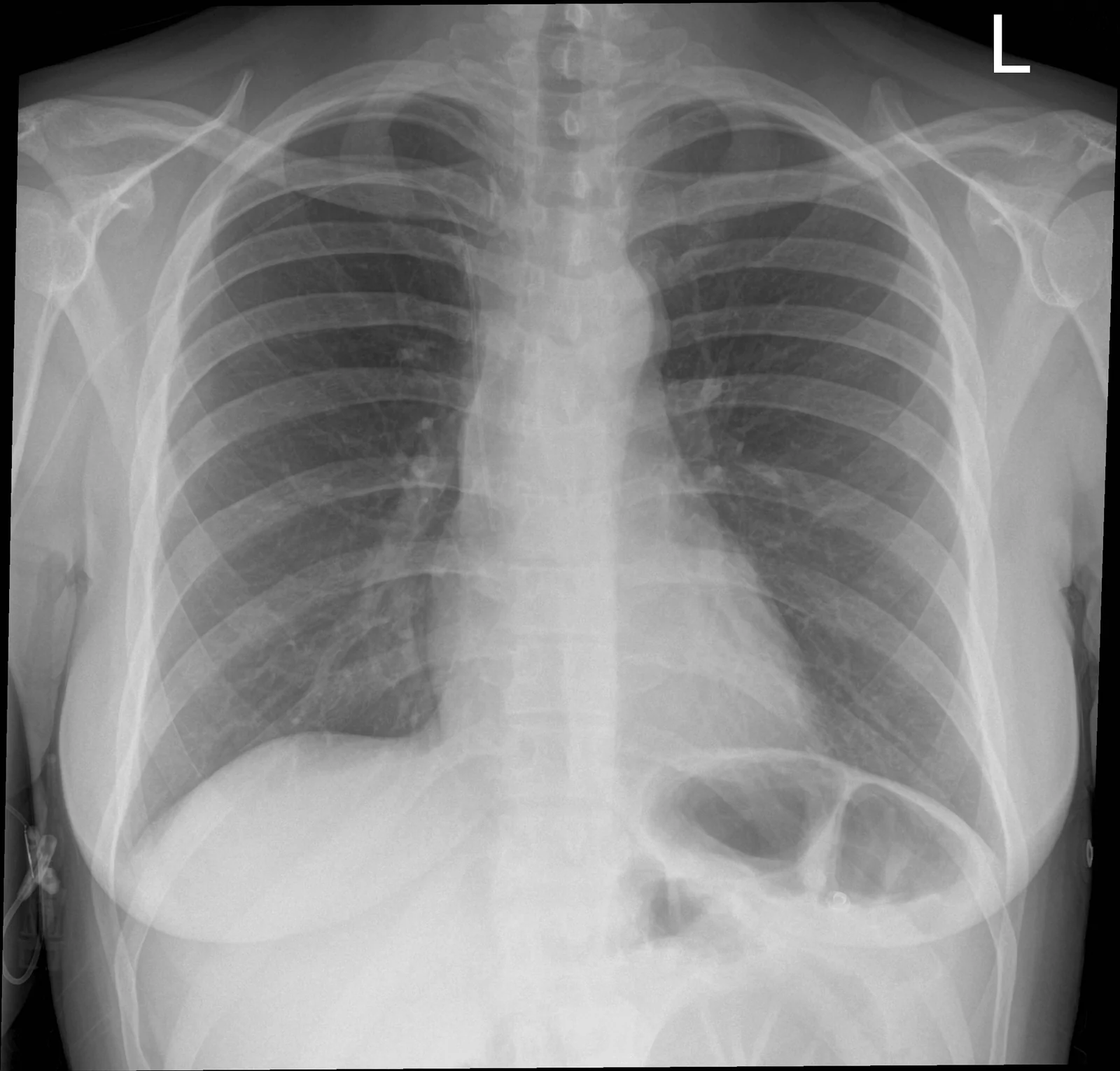
Post-pericardiocentesis CXR showing interval improvement in cardiomegaly/pericardial effusion appearance.

Due to the severity of the disease, treatment was initiated urgently with one cycle of rituximab, cyclophosphamide, hydroxydaunorubicin (doxorubicin), vincristine (Oncovin), and prednisone (R-CHOP) before escalation to dose-adjusted rituximab, etoposide, prednisone, vincristine (Oncovin), cyclophosphamide, and doxorubicin (R-EPOCH) following multidisciplinary team discussion. Goserelin was commenced for ovarian protection prior to subsequent chemotherapy cycles. During treatment, the patient developed coronavirus disease 2019 (COVID-19), which was successfully managed with sotrovimab.

Interim positron emission tomography-computed tomography (PET-CT) performed after two cycles demonstrated an excellent metabolic response, with residual low-grade uptake corresponding to a Deauville score of 4. End-of-treatment PET-CT subsequently confirmed complete metabolic remission.

At follow-up, the patient remained clinically well, with complete resolution of cardiorespiratory symptoms and no evidence of recurrent disease. She continues under regular haematology follow-up with surveillance echocardiography and prophylactic antimicrobial therapy because of persistent lymphopenia.

## Discussion

This case highlights an atypical and potentially life-threatening presentation of PMBCL with a large pericardial effusion and impending cardiac tamponade in a young, previously healthy woman. PMBCL is an uncommon subtype of large B-cell lymphoma that predominantly affects young adults and typically presents with a bulky anterior mediastinal mass. Clinical manifestations are often related to local compression and may include dyspnoea, chest pain, cough, and symptoms associated with superior vena cava obstruction [1-5]. Although cardiovascular involvement is uncommon, pericardial effusion and cardiac tamponade have been reported as rare presenting manifestations of PMBCL [3].

Cardiac tamponade occurs when accumulation of pericardial fluid increases intrapericardial pressure, impairing ventricular filling and reducing cardiac output. In patients with malignancy, pericardial effusion may develop through direct tumour infiltration, lymphatic obstruction, impaired venous drainage, or inflammatory mechanisms [6]. The clinical presentation can vary depending on the rate and volume of fluid accumulation. Although hypotension, elevated jugular venous pressure, and muffled heart sounds are classically associated with cardiac tamponade, these findings may be absent, particularly when fluid accumulates gradually. Consequently, preserved blood pressure should not be interpreted as evidence against clinically significant tamponade.

In this patient, despite a large pericardial effusion requiring drainage of approximately 1.5 L, there was no significant hypotension, and the jugular venous pressure was not clearly elevated. Typical clinical features of significant pericardial effusion include dyspnoea, fatigue, peripheral oedema, and signs of systemic venous congestion; in this case, several of these features were present, while hypotension and clear jugular venous distension were absent. The combination of progressive dyspnoea, orthopnoea, peripheral oedema, cardiomegaly, bilateral pleural effusions, and low-voltage QRS complexes provided important diagnostic clues. Low-voltage QRS complexes in the presence of cardiomegaly should raise suspicion for significant pericardial effusion, particularly when accompanied by symptoms suggestive of impaired cardiac filling. Echocardiography is central to the assessment of suspected cardiac tamponade, while cross-sectional imaging can provide additional information regarding the underlying cause and extent of mediastinal disease [6].

The initial clinical presentation was non-specific and included features that could reasonably suggest pulmonary embolism, pericarditis, or myocarditis. The elevated D-dimer further supported the need to investigate for pulmonary embolism. However, CT pulmonary angiography identified the underlying anterior mediastinal mass together with a large pericardial effusion and radiological features of tamponade. This highlights the importance of maintaining a broad differential diagnosis when evaluating young patients with unexplained cardiorespiratory symptoms, particularly when initial investigations reveal abnormalities that cannot be fully explained by the presumed diagnosis.

Emergency pericardiocentesis was critical to stabilising the patient and resulted in significant symptomatic improvement. Management of malignant pericardial effusion requires both treatment of the immediate haemodynamic complication and definitive management of the underlying malignancy [6]. The rapid clinical improvement following drainage in this case demonstrates the importance of recognising significant pericardial disease before overt haemodynamic collapse occurs.

Following stabilisation, the patient underwent lymphoma-directed therapy. Contemporary management of PMBCL has evolved considerably, with dose-adjusted rituximab, etoposide, prednisone, vincristine, cyclophosphamide, and doxorubicin (DA-R-EPOCH) established as an effective treatment approach associated with high rates of disease control and long-term remission [7]. In this case, R-CHOP (rituximab, cyclophosphamide, doxorubicin, vincristine, and prednisone) was initially administered because of the severity of the presentation and the need for urgent tumour cytoreduction, followed by escalation to DA-R-EPOCH following multidisciplinary discussion. The patient subsequently achieved complete metabolic remission on end-of-treatment PET-CT.

The atypical nature of cardiac tamponade as an initial manifestation of PMBCL has been described in previous reports. Such cases emphasise that significant pericardial involvement may occur in the absence of the classical clinical findings of tamponade [3]. Cardiac tamponade has also been reported as an initial manifestation of other lymphomas, including Hodgkin lymphoma, demonstrating that malignant pericardial involvement should remain within the differential diagnosis of unexplained pericardial effusion [8]. More recent reports continue to highlight the potential for PMBCL to present with cardiac tamponade, reinforcing the importance of early recognition of this rare complication [9].

The patient's interim PET-CT demonstrated an excellent metabolic response, with residual low-grade uptake corresponding to a Deauville score of 4, followed by complete metabolic remission at the end of treatment. This favourable response is consistent with the high treatment responsiveness of PMBCL when appropriate lymphoma-directed therapy is initiated [2,7]. The case therefore illustrates that even patients presenting with severe and potentially life-threatening complications can achieve excellent outcomes when the underlying malignancy is rapidly recognised and treated.

Fertility preservation and ovarian protection are also important considerations in young patients receiving intensive chemotherapy. The use of goserelin in this patient reflects the importance of addressing reproductive considerations at the outset of treatment, particularly in young women with potentially curable lymphoma. Such considerations should form part of the multidisciplinary management of PMBCL alongside disease control and prevention of treatment-related complications.

Overall, this case demonstrates that cardiac tamponade may be an unusual initial manifestation of PMBCL and can occur without hypotension, marked jugular venous distension, or other classical signs. Clinicians should maintain a high index of suspicion when young patients present with unexplained dyspnoea, cardiomegaly, bilateral pleural effusions, or low-voltage ECG changes. Early echocardiographic and cross-sectional imaging, prompt pericardial drainage when clinically indicated, and rapid initiation of definitive lymphoma therapy are essential to prevent deterioration and optimise outcomes.

## Conclusions

Cardiac tamponade is a rare but potentially life-threatening complication of primary mediastinal B-cell lymphoma and may present without the classical clinical features of tamponade. In young patients with unexplained dyspnoea, cardiomegaly, bilateral pleural effusions, or low-voltage QRS complexes, significant pericardial effusion should be considered even in the absence of hypotension or obvious jugular venous distension. Early echocardiographic and cross-sectional imaging can facilitate diagnosis, while prompt pericardial drainage and timely initiation of lymphoma-directed therapy are essential. This case highlights the importance of maintaining a high index of suspicion for malignant pericardial involvement and recognising atypical presentations of cardiac tamponade to prevent potentially fatal deterioration.
